# Transosseous Suturing for the Correction of Juvenile Hallux Valgus: A Preliminary Case Series Study

**DOI:** 10.3390/medicina58111679

**Published:** 2022-11-19

**Authors:** Wei-Chen Hung, Shu-Hsin Yao, Ting-Ming Wang, Chun-Ho Chen

**Affiliations:** 1 Department of Orthopedics, Ditmanson Medical Foundation Chia-Yi Christian Hospital, Chiayi 60002, Taiwan; 2 Department of Orthopedic Surgery, National Taiwan University Hospital, Taipei 100229, Taiwan; 3 School of Medicine, National Taiwan University, Taipei 10617, Taiwan

**Keywords:** juvenile hallux valgus, transosseous suturing technique, medial capsuloligamentous augmentation, Fiberwire

## Abstract

*Background and Objectives*: Many treatment modalities are available for juvenile hallux valgus. However, all of them have some disadvantages. Therefore, we developed a transosseous suturing procedure. *Materials and Methods*: Six patients (seven feet) with juvenile hallux valgus received transosseous suturing procedure. Clinical and radiological examinations were performed preoperatively and postoperatively. All patients underwent the soft tissue release procedure, followed by transosseous suturing with Fiberwire (in which sutures are anchored with mini plates). *Results*: The mean IMA and HVA decreased from 15.6° ± 2.6° to 7.3° ± 1.1° and 39.2° ± 3.3° to 12.5° ± 3.1°, respectively. Corrections achieved in the IMA and HVA of all patients were maintained through the last follow-up. The mean American Orthopedic Foot & Ankle Society score improved from 53.3 ± 3.5 to 86.9 ± 4.7 points. *Conclusions*: Based on these preliminary data, the transosseous suturing technique demonstrated satisfactory results and apparent improvements in the IMA and HVA without early complications.

## 1. Introduction

Hallux valgus is a common foot problem that causes pain and disability. This condition is relatively uncommon in children and is also known as juvenile or adolescent bunion, metatarsus primus varus, and metatarsus primus adductus. Patients aged < 18 years with a hallux valgus angle (HVA) of >20° and an intermetatarsal angle (IMA) of >10° are diagnosed as having juvenile hallux valgus (JHV) [1,2]. A classification of JHV is also reported by Philip depending on HVA as normal (HVA: 0–15°), mild–moderate (HVA: 15–35°), and severe (HVA: >35°) [3].

The etiology and pathogenesis of this deformity are complex and have not been understood clearly. A study reported that maternal transmission, neuromuscular conditions, and idiopathic disease are the etiological factors [4]. Symptoms may include pain and soreness at the base of the great toe, bunion deformities with difficulty wearing footwear, and unsatisfactory cosmesis.

The treatment of hallux valgus deformities in juvenile patients is still controversial due to skeletal immaturity in children [5]. For asymptomatic patients with mild deformity, conservative treatment is indicated. Operative intervention is usually required for patients with severe painful deformity [6,7]. Traditionally, osteotomy is avoided in skeletally immature patients to prevent growth problems. Many surgeons usually choose soft-tissue surgery to correct juvenile hallux valgus; however, it has a relatively high recurrence rate, and the IMA cannot be corrected through soft-tissue surgery alone [8]. Theoretically, metatarsal osteotomy can apparently improve the IMA; thus, some authors have recommended osteotomy for patients with open physis. However, studies have reported high rates of complications such as nonunion and malunion as well as recurrence (20–40%) [9,10,11,12]. Hemiepiphysiodesis is another proposed treatment, but it can result in poor correction strength. Furthermore, the hemiepiphysiodesis procedure does not involve changes in the ligament structure [13]. Therefore, some surgeons prefer osteotomy with or without soft-tissue surgery until the patient reaches skeletal maturity [14].

To prevent complications caused by osteotomy, Mini TightRope was introduced [15]. This is a suture button system used to approximate the first and second metatarsals and achieve IMA correction. Although this procedure satisfactorily improved the IMA, some severe complications were reported, including the fracture of the metatarsal, followed by fixation failure [16].

To prevent the aforementioned complications, we developed a transosseous suturing technique by reinforcing the intermetatarsal ligament and medial collateral ligament with Fiberwire and a miniplate. In this study, we investigated the efficacy of this new method in treating juvenile hallux valgus.

## 2. Materials and Methods

This study was approved by Ditmanson Medical Foundation Chia-Yi Christian Hospital ethical review board (approval no. CYCHIRB 2021063). Six symptomatic children (seven feet) with severe juvenile hallux valgus who were treated with the transosseous suturing technique in our institution from 2017 to 2020 were selected. All children complained of significant pain and obvious hallux deformity with failed conservative treatment, including shoe modification or orthotics for several years without apparent improvement, and subsequently underwent surgery. All of them were girls. Their average age was 12.9 ± 1.7 (range: 11 to 15) years. The mean follow-up period was 32.1 ± 15.6 (range: 12 to 50) months. Radiography and functional scores were regularly obtained during follow-up (Table 1).

We obtained all the radiographs of the feet in standard weight-bearing view preoperatively, non-weight-bearing view at 2-week postoperatively, and weight-bearing view at 3-month, 6-month, and 1-year postoperatively. We measured the HVA and IMA. For functional assessments, we used the American Orthopedic Foot & Ankle Society (AOFAS) hallux metatarsophalangeal–interphalangeal score for evaluation [17]. This assessment system includes nine questions under three categories: pain (40 points), function (50 points), and alignment (10 points). The total score ranges from 0 to 100 points. All patients were evaluated preoperatively and at the final follow-up visit. The satisfaction was determined at the 12-month follow-up, and all patients were asked whether they would have the same surgery again given similar conditions.

**Figure 1:**
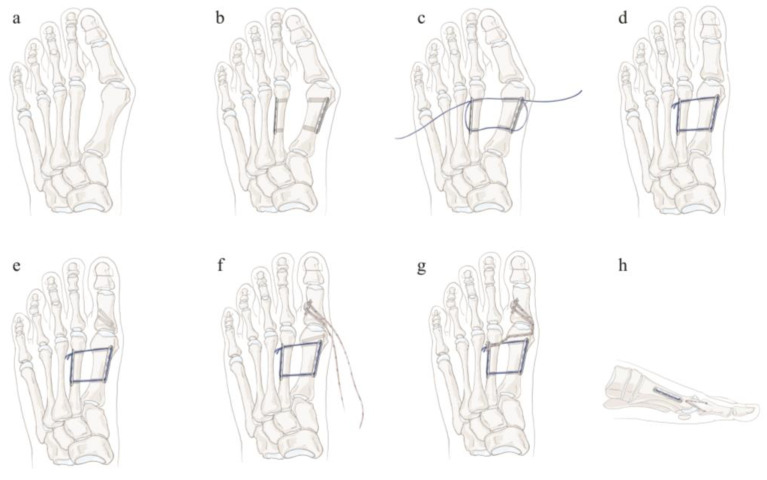
Illustration of the transosseous suturing technique. (**a**) Prepare two bone tunnels by drilling from the first metatarsal to the second metatarsal. (**b**) Subsequently, place two mini plates on the medial side of the first metatarsal bone and lateral side of the second metatarsal bone. (**c**) Pass Fiberwire through the two mini plates and bone tunnels at the metatarsal bones thrice. (**d**) After Fiberwire is set up properly, tighten it and anchor the suture on the plate. (**e**) If augmentation of the medial capsuloligamentous structure is required for a patient, drill two bone tunnels at the first proximal phalanx and one bone tunnel at the medial collateral ligament insertion site on the first metatarsal. (**f**) For medial capsuloligamentous augmentation, two Fiberwires were tied on the washer applied at the lateral side of the first proximal phalanx and passed through the dorsal- and plantar-side bone tunnels, respectively. (**g**) Then, two Fiberwires were passed through the first metatarsal at the medial collateral ligament insertion site and the second metatarsal distal bone tunnel. After passing through Fiberwires, the suture was anchored on the plate. (**h**) Lateral view of the final appearance of the structure.

**Figure 2:**
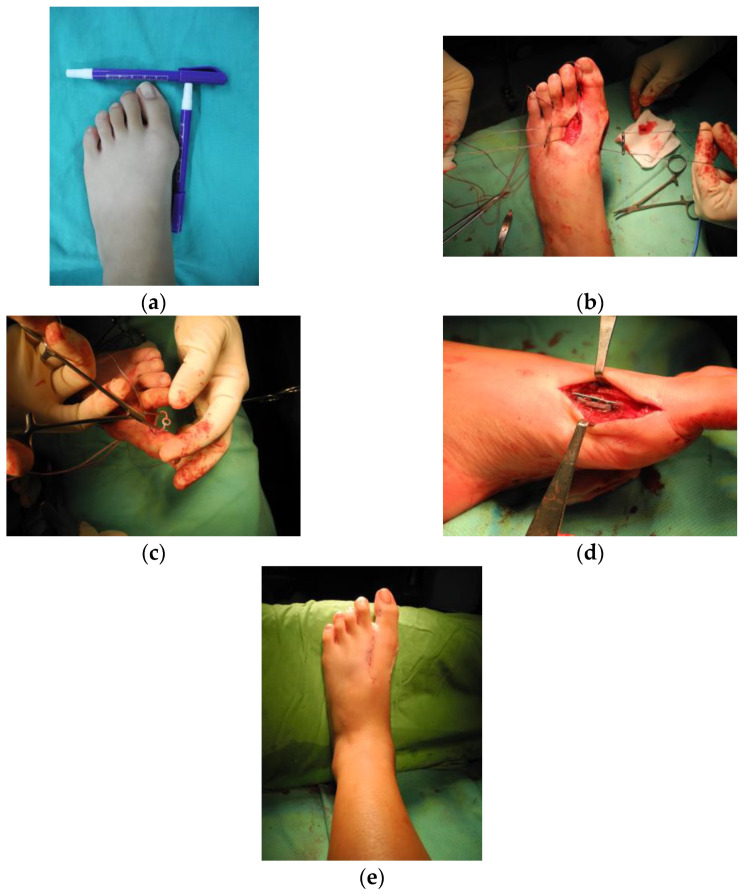
Surgical photos showing some steps in the transosseous suturing technique. (**a**) Preoperative general appearance of the hallux valgus. (**b**) Mini plates applied at the first and second metatarsals through the wire. (**c**) Medial capsuloligamentous augmentation for anchoring Fiberwire with the washer. (**d**) Lateral view of the final position of the mini plate parallel to the metatarsal. (**e**) Postoperative general appearance after the correction of hallux valgus.

For the procedure, the patient was placed in the supine position under general anesthesia. A skin incision was made along the first web space, and the attachment of the adductor halluces muscle was released. The articular capsule was opened through the transection of the lateral collateral ligament, and the lateral metatarsosesamoid ligament was released from the first metatarsal–phalangeal (MTP) joint. Subsequently, another longitudinal skin incision was made along the medial site, and capsulotomy was performed. We prepared bone tunnels on the first and second metatarsals under fluoroscopic guidance (Figure 1a). Two Fiberwire sutures (Arthrex, Naples, FL, USA) were passed from the first metatarsal bone to the second metatarsal bone thrice under careful protection of neurovascular structure from 1–2 metatarsal interspace wound and anchored using two mini plates (LCP compact foot, Depuy Synthes, West Chester, PA, USA) at the medial side of the first metatarsal bone and lateral side of the second metatarsal bone (Figure 1b–d). The 1st and 2nd metatarsal bones were held in proper alignment with a bone clamp while we were passing and tying the sutures. The tension of the device was carefully rechecked manually after releasing the bone clamp to make sure that the suture and tie were strong enough. For selected patients (HVA > 40°), we added the additional sutures to augment the medial capsuloligamentous structure at the first metatarsophalangeal joint (Figure 1e–h). Finally, capsulorrhaphy over the medial first MTP joint was performed, and the wound was closed in layers (Figure 2). Generally, orthoses were used for postoperative protection in adult hallux valgus surgeries. However, it is not easy for a pediatric patient to properly follow the postoperative instruction. Thus, we routinely applied short leg casts for pediatric patients and advised non-weight bearing for 6 weeks. Weight-bearing radiographs were obtained at 3 months, 6 months, and 1 year.

The generalized estimating equation method was used to compare the repeated measures of AOFAS, HVA, and IMA for different time points, and all analyses were performed using IBM SPSS Statistics (Version 21.0.; IBM Corp., Armonk, NY, USA).

## 3. Results

### 3.1. Radiographic Outcomes

**Figure 3:**
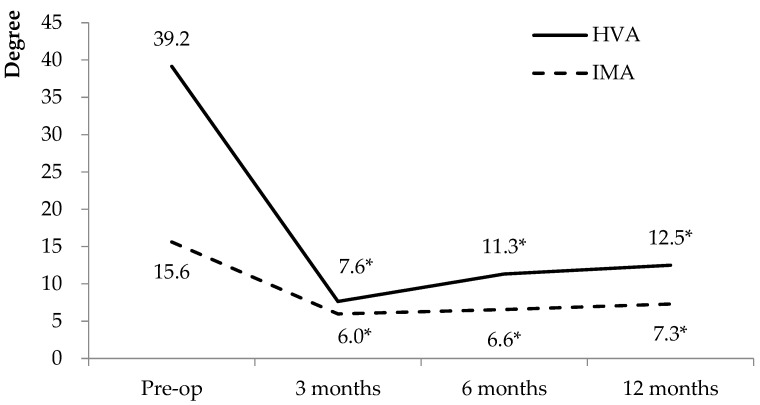
Radiographic outcomes. * Compared with baseline parameters, *p* < 0.001. HVA: hallux valgus angle, IMA: intermetatarsal angle.

For radiographic parameters, the weight-bearing radiograph at 3 months, 6 months, and 1 year postoperatively were recorded. The mean HVA preoperatively for all the feet was 39.2° ± 3.3°, which significantly decreased (*p* < 0.001) to 12.5° ± 3.1°at the postoperative 1-year follow-up. The mean improvement in the HVA was 26.7°. For the IMA, the preoperative mean angle was 15.6° ± 2.6°, which significantly decreased (*p* < 0.001) to 7.3° ± 1.1° at the postoperative 1-year follow-up. The mean correction of the IMA was 8.3° (Figure 3).

### 3.2. Functional Outcomes

**Figure 4:**
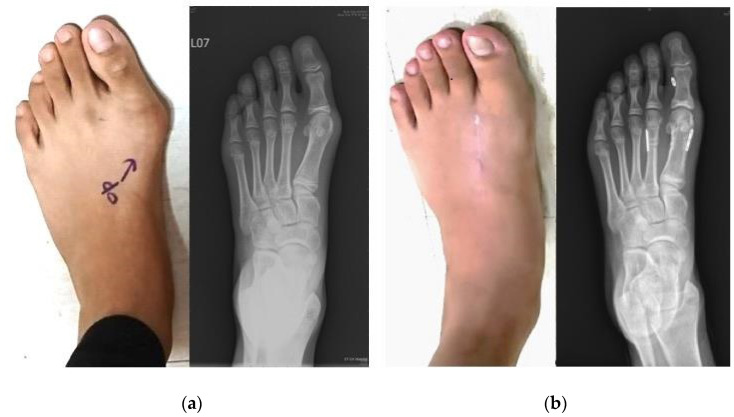
Clinical appearance and radiography (anteroposterior view) of the left foot. (**a**) Preoperative standing weight-bearing view and (**b**) 1-year follow-up postoperative standing weight-bearing view.

Clinically, the AOFAS hallux metatarsophalangeal interphalangeal scores significantly improved (*p* < 0.001) from 53.3 ± 3.5 points preoperatively to 86.9 ± 4.7 points at the postoperative 1-year follow-up. All patients were satisfied with surgical results in terms of both cosmetic appearance and functional abilities and returned to school or daily activities (Figure 4). All of the patients agreed to undergo the same procedure given similar conditions. No known complication was noted, and no patient needed revision surgery for poor outcomes.

## 4. Discussion

Juvenile hallux valgus is a relatively uncommon but concerning deformity in children. The indication and timing of operation are controversial [18]. Traditionally, indications for surgery are deformity worsening or persistent significant pain [6]. Surgery is usually performed after skeletal maturity [1]. The treatment is variable, and the most commonly used methods include soft-tissue surgery, osteotomy, hemiepiphysiodesis, and suture button device procedure.

A soft-tissue surgery without any osteotomy is indicated in immature bones to reduce the possibility of physeal injury. However, some studies have reported a high rate of hallux valgus recurrence and only slight improvement in the HVA without any change in the IMA with soft-tissue surgery alone [19]. This may be due to the general laxity and elasticity of these tissues in juvenile patients [20]. Hence, these patients may have residual deformity after surgery with a high risk of revision surgery.

For bony realignment with osteotomy correction, although more correction degrees can be achieved, several complications have been reported in many studies. These complications include first metatarsal head avascular necrosis (AVN), malunion (shortening, rotation, or dorsiflexion of the first ray), nonunion, delayed union, and first MTP joint stiffness [6,21,22]. These complications make it difficult to predict osteotomy outcomes. Therefore, with so many uncertain factors of osteotomy correction, avoiding osteotomy treatment in juveniles has become the goal of pediatric surgery.

Hemiepiphysiodesis and the suture button device procedure were proposed recently as the new methods for correcting hallux valgus without osteotomy [23]. However, a recent study revealed that the correction potential of hemiepiphysiodesis is not as powerful as that of osteotomy [13]. For the suture button device, although the power of correction is stronger than that of hemiepiphysiodesis, some complications were noted, including hardware failure and metatarsal fracture. In addition, the bones of children are smaller than those of adults, thus increasing the risk of metatarsal fracture in juvenile patients [16,24].

**Figure 5:**
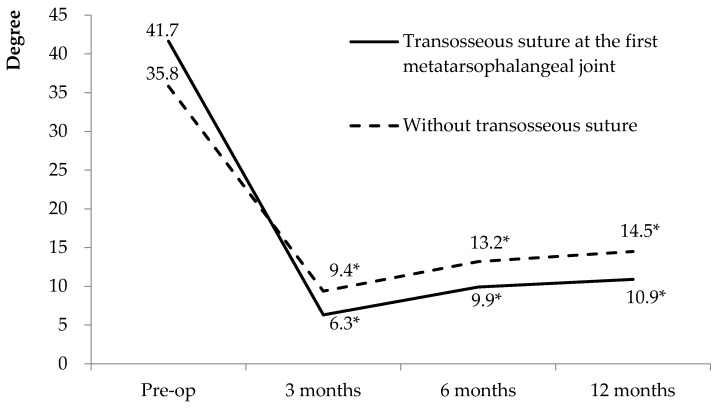
Comparison of the HVA between those with and without medial capsuloligamentous augmentation at the first metatarsophalangeal joint. * Compared with the baseline parameters, *p* < 0.001.

To prevent these severe complications, we developed a transosseous suturing technique that significantly reduced the IMA and HVA after surgery in this study. Compared with a suture button device procedure, our method, transosseous suturing, increases the contact area between the implant and the bone surface. Thus, the stress is equally distributed to the large area of the bone. Theoretically, it reduces the risk of metatarsal fracture. All seven feet had satisfactory correction for juvenile hallux valgus without any complication of fracture or tendon rupture. A significant decrease in the IMA and HVA was observed from the preoperative status to the last follow-up. Furthermore, a mild increase in the IMA and HVA at 2 weeks to 6 months postoperatively may be due to the transition from non-weight-bearing to weight-bearing at 2 weeks postoperatively, which increases the stress on the transosseous suturing device. Deformity correction and functional outcomes were satisfactory in all patients. A shortcoming of this operation is that the incision wound may be relatively larger than that in the suture button device technique. Four of the seven feet that underwent medial collateral ligament reconstruction exhibited more improvement in the HVA at the final follow-up (Figure 5). Lui et al. reported that the medial capsuloligamentous structure plays a crucial role in the stability of the first metatarsophalangeal joint [25]. According to this study, and on the basis of our findings, we recommend all patients undergo medial capsuloligamentous augmentation to achieve a more favorable cosmetic outcome and reduce recurrence risk.

There is a limitation of our technique. During the short-term follow-up, a trend of slightly increasing IMA over time can be observed. The angles seem to become stabilized 6−12 months after surgery (Figure 3 and Figure 5). Although the IMA did not reach the diagnostic criteria of HV, there will still be a concern that the deformity will recur in the future. We did not find any sudden increase of the IMA or HVA, indicating failure of the device in our short-term result. However, broken suture material, loosening of the suture ties, or fracture of the bones might be the major complications of this technique. A long-term follow-up result with continuous monitoring of the IMA and HVA will be needed to confirm the advantage of this procedure.

For our limited experience and the small sample size, no perioperative complications were noted, including wound infection, over-correction, or neurovascular injury. However, for this technique, there are some things we need to pay attention to. The first one is the neurovascular bundle injury. To avoid this complication, we will directly see the wire passing through and give well protection from 1–2 metatarsal interspace wounds during the procedure. Another concern is metatarsal head avascular necrosis (AVN). The periosteum may get hurt, and blood supply may be influenced during the plate insertion. However, since no osteotomy was performed, we can retain the endosteal blood supply and ensure the metatarsal head with adequate blood supply.

This study has some limitations. First, there is no control group in our study for comparing our treatment with other treatments for juvenile hallux valgus. Additionally, as a case series study, the sample size is really small to make a strong conclusion. Further, this is a preliminary report with a relatively short-term follow-up period. Although short-term results showed a favorable outcome, a larger number of cases with a longer follow-up period will be needed to investigate the long-term results and the complications.

## 5. Conclusions

The management of pediatric hallux valgus is variable. The transosseous suturing method demonstrated satisfactory results, apparent improvements in IMA and HVA, and no early complications. In our limited experience, the transosseous suturing method may be a satisfactory choice for the correction of juvenile hallux valgus.

## Figures and Tables

**Table 1:** Demographic characteristics.

Patient No.	Sex	Age (Years)	Side	Follow-Up (Months)	AOFAS (0–100)	
					Pre-Op	Final Follow-Up
1	F	15	L	35.0	59	85
2	F	13	L	42.2	54	88
3	F	11	L	48.2	54	93
4	F	12	L	21.5	54	83
4	F	13	R	15.9	54	80
5	F	15	L	50.0	49	87
6	F	11	L	12.0	49	92

## Data Availability

The data presented in this study are available on request from the corresponding author.
